# Supplementary material for: Calreticulin del52 and ins5 knock-in mice recapitulate different myeloproliferative phenotypes observed in patients with MPN
Source: Nat Commun. 2020 Sep 28;11:4886. doi: 10.1038/s41467-020-18691-3 (PMC7522233; doi:10.1038/s41467-020-18691-3)
Supplement: Supplementary file 1 — Supplementary Information [file 41467_2020_18691_MOESM1_ESM.pdf]

Calreticulin del52 and ins5 knock-in mice recapitulate different myeloproliferative phenotypes observed in patients with MPN

Benlabiod *et al.*

Supplementary material

**Name of primer** (F for forward/R for reverse; use on genomic DNA)	5' to 3' sequence
**a** (F; *del52* KI genotyping)*	CACACCCTGCTGCCCCAC
**b** (R; *del52* KI genotyping)*	CCTTGGCTTGGCCAGGG
**c** (F; *ins5* KI genotyping)	CAGCCAGGCAGGCTGGTCTTG
**d** (R; *ins5* KI genotyping)	GTGTGGTAGGACATGCCTTAGATCC
**p1** (F; SCL-CreER^T genotyping)	TCGATGCAACGAGTGATGAG
**p2** (R; SCL-CreER^T genotyping)	GGTCGGCCGTCAGGGACAA
**p3** (F; SCL-CreER^T genotyping)	AGCATGCTCTTTTCCAGCAT
**p4** (R; SCL-CreER^T genotyping)	CTCAGGCTGGCCTAAAACTG
**muCALR_wt_ex8_F** (F; wt mouse *Calr* genotyping)†	GTCAAGTCCGGGACAATCTT
**muCALR_wt_onT2_R** (R; wt mouse *Calr* genotyping)†	TCATCCTCTTTATCCTCAGCT
**muCALR_wt_int8-9_F** (F; *ins5* KI allele)†	CATCTGGGAGGACTGGCAGAC
**chimCALR_ins5_R** (R; *ins5* KI allele)†	CTCCGACAATTATCCTCAG

Supplementary Table 1. List of the primers used in the study. * See Fig. 1a for position of the primers on the gene. † See Supplementary Fig. 11 for position of the primers on the genes and the genotyping PCR strategy.

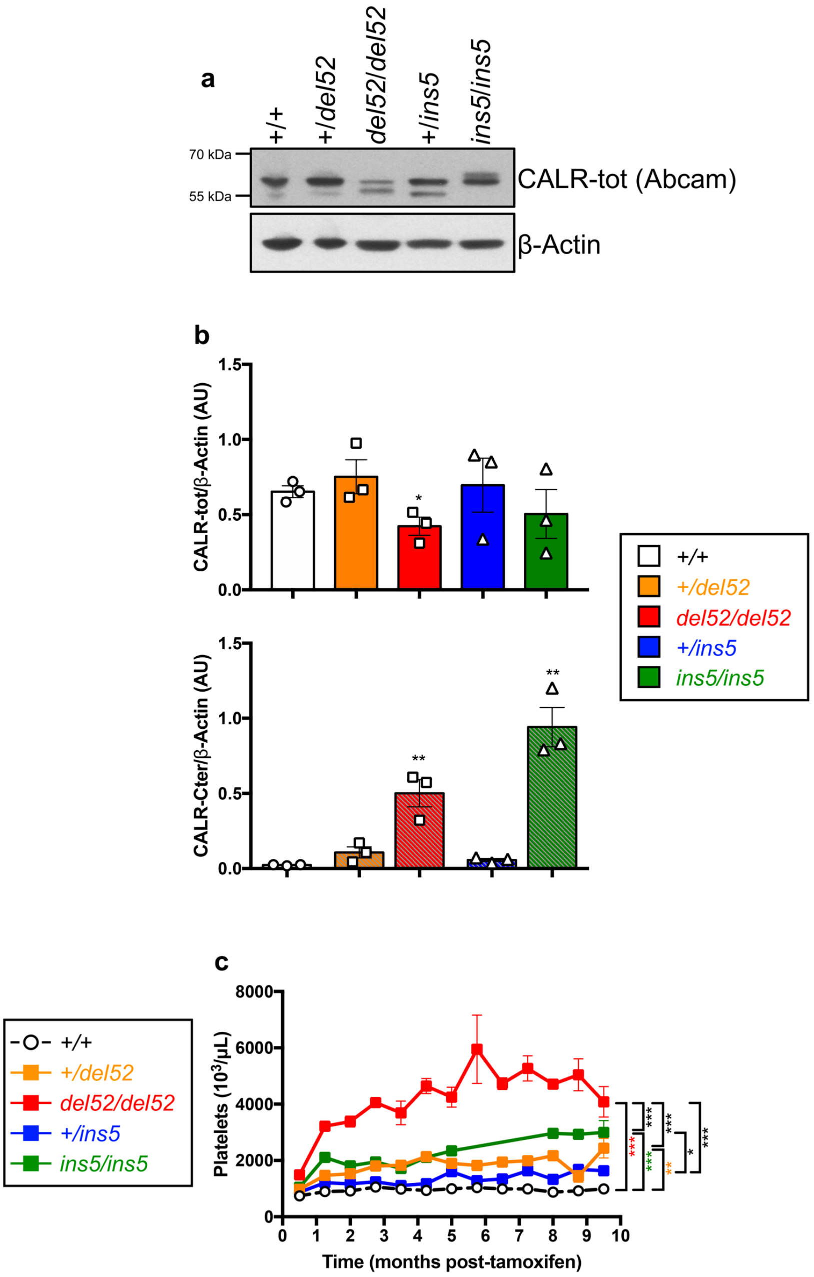

Supplementary Fig. 1. CALR protein expression levels and platelet counts of *CALR del52* and *CALR ins5* KI mice. a Lin$^-$ cells isolated from BM of wt (+/+), heterozygous (+/*del52* or +/*ins5*) and homozygous (*del52/del52* and *ins5/ins5*) *CALR*-mutated KI mice were analyzed by Western blot for expression of CALR using an Ab directed against amino acids 50-150, common to both wt and mutant CALR (CALR-tot, Abcam). ß-Actin was used as loading control. Data are representative of a typical experiment that was reproduced using at least 3 independent mice for each genotype. **b** Data represent the quantification of n=3 independent

Western blot experiments using CALR-tot and CALR-Cter (specific of the mutated C-terminus) Abs with the ImageJ software and are expressed as means±SEM. In the figure, white is used for +/+, orange for *+/del52*, red for *del52/del52*, blue for *+/ins5* and green for *ins5/ins5* genotypes. Statistical analysis was performed with a two-sided parametric t-test (*p=0.0329; **p=0.0061 (+/+ *vs. del52/del52*), 0.0022 (+/+ *vs. ins5/ins5*)). **c** Comparison of the time-dependent increase in blood platelet levels of heterozygous and homozygous *del52* and *ins5* KI mice after tamoxifen induction. Data are the means±SEM (n=4-18 +/+ mice (open circles with connecting black dashed line), n=3-20 *+/del52* (orange squares and solid line), n=3-14 *del52/del52* (red squares and solid line), n=4-20 *+/ins5* (blue squares and solid line), n=5-9 *ins5/ins5* (green squares and solid line) with n representing the number of individual mouse depending on time of sampling during the course of the experiment, as indicated in the Source Data file). Statistical significance was determined using ANOVA with Dunnett's multiple comparisons test: *p=0.0106; **p=0.0061; ***p<0.0001. Source data are provided as a Source Data file.

a

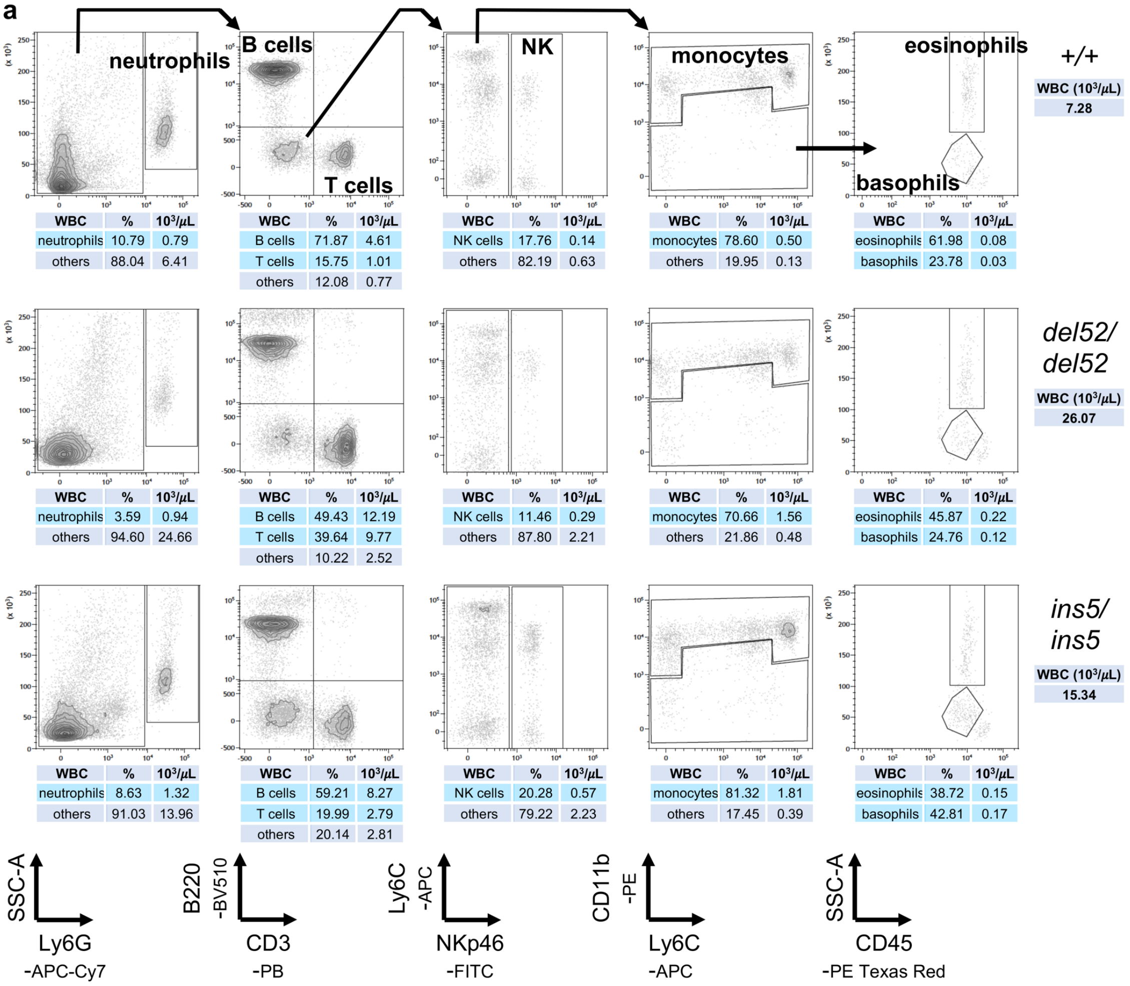

+/+ (WBC $10^3/\mu L$: 7.28)

WBC	%	$10^3/\mu L$
neutrophils	10.79	0.79
others	88.04	6.41

WBC	%	$10^3/\mu L$
B cells	71.87	4.61
T cells	15.75	1.01
others	12.08	0.77

WBC	%	$10^3/\mu L$
NK cells	17.76	0.14
others	82.19	0.63

WBC	%	$10^3/\mu L$
monocytes	78.60	0.50
others	19.95	0.13

WBC	%	$10^3/\mu L$
eosinophils	61.98	0.08
basophils	23.78	0.03

del52/del52 (WBC $10^3/\mu L$: 26.07)

WBC	%	$10^3/\mu L$
neutrophils	3.59	0.94
others	94.60	24.66

WBC	%	$10^3/\mu L$
B cells	49.43	12.19
T cells	39.64	9.77
others	10.22	2.52

WBC	%	$10^3/\mu L$
NK cells	11.46	0.29
others	87.80	2.21

WBC	%	$10^3/\mu L$
monocytes	70.66	1.56
others	21.86	0.48

WBC	%	$10^3/\mu L$
eosinophils	45.87	0.22
basophils	24.76	0.12

ins5/ins5 (WBC $10^3/\mu L$: 15.34)

WBC	%	$10^3/\mu L$
neutrophils	8.63	1.32
others	91.03	13.96

WBC	%	$10^3/\mu L$
B cells	59.21	8.27
T cells	19.99	2.79
others	20.14	2.81

WBC	%	$10^3/\mu L$
NK cells	20.28	0.57
others	79.22	2.23

WBC	%	$10^3/\mu L$
monocytes	81.32	1.81
others	17.45	0.39

WBC	%	$10^3/\mu L$
eosinophils	38.72	0.15
basophils	42.81	0.17

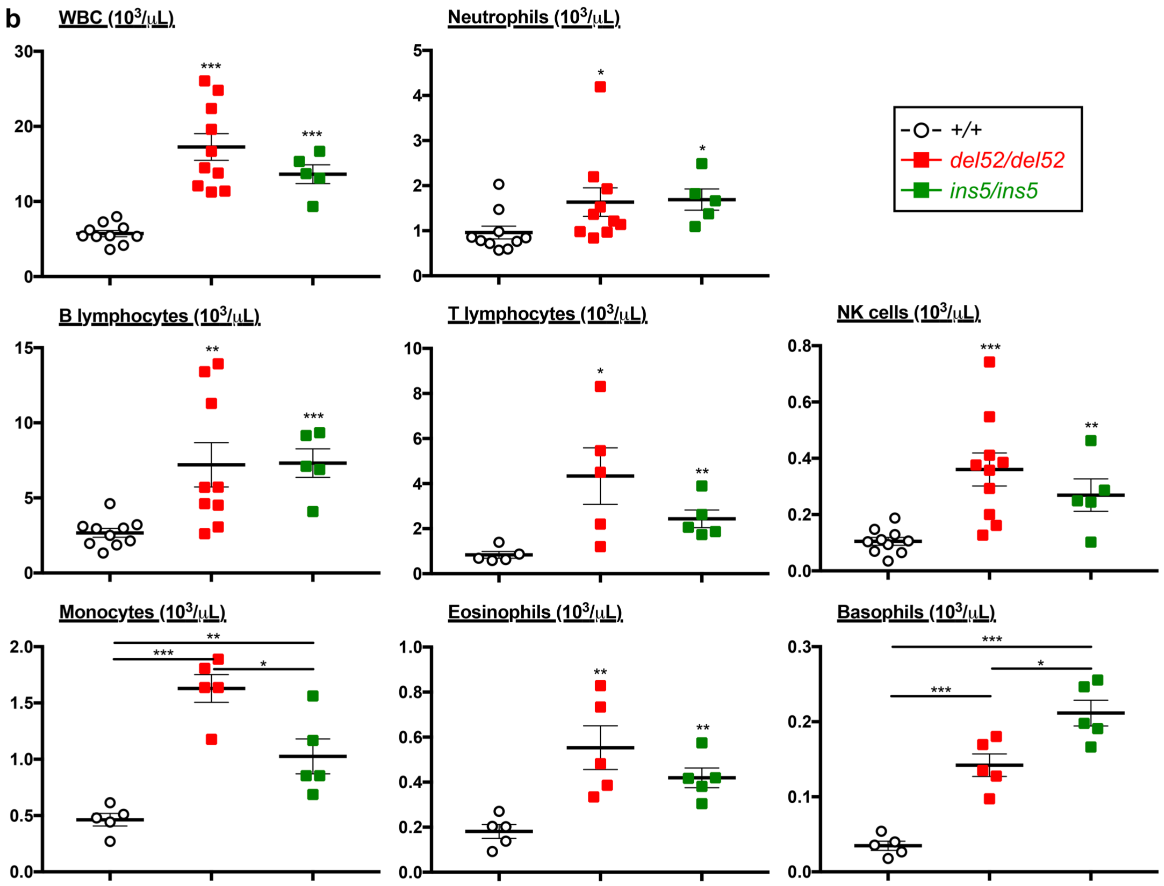

Supplementary Fig. 2. Characterization of WBC content of *CALR del52* and *CALR ins5* KI mice. a Gating strategy used to analyze WBC composition by flow cytometry from blood of ≥10-month-old +/+ (open circles), *del52/del52* (red squares) and *ins5/ins5* (green squares) KI mice. Absolute numbers of polynuclear neutrophils ($Ly6G^+$), B ($B220^+$) and T ($CD3^+$) lymphocytes, natural killer cells ($NKp46^+$), monocytes ($CD11b^+$ and $Ly6C^{-/+}$) and finally eosinophils ($CD45^{high}SSC^{high}$) and basophils ($CD45^{low}SSC^{low}$) were measured by flow cytometry and **b** expressed as means±SEM (n=5 independent mice except n=10 for +/+ and *del52/del52* genotypes when analyzing WBC, neutrophils, B lymphocytes and NK cells). Two-sided both parametric t-test and nonparametric Mann-Whitney test (only for Neutrophils) were used: *p=0.0147 (+/+ *vs. del52/del52*, neutrophils), 0.0193 (+/+ *vs. ins5/ins5*, neutrophils), 0.0241 (+/+ *vs. del52/del52*, T lymphocytes), 0.0157 (*del52/del52 vs. ins5/ins5*, monocytes), 0.0156 (*del52/del52 vs. ins5/ins5*, basophils); **p=0.0056 (+/+ *vs. del52/del52*, B

lymphocytes), 0.0052 (+/+ *vs. ins5/ins5*, T lymphocytes), 0.0025 (+/+ *vs. ins5/ins5*, NK cells,), 0.0092 (+/+ *vs. ins5/ins5*, monocytes), 0.0065 (+/+ *vs. del52/del52*, eosinophils), 0.0022 (+/+ *vs. ins5/ins5*, eosinophils, monocytes); ***p<0.0001 (WBC, B lymphocytes, basophils for +/+ *vs. ins5/ins5*), 0.0005 (+/+ *vs. del52/del52*, NK cells), 0.0002 (+/+ *vs. del52/del52*, basophils). Source data are provided as a Source Data file.

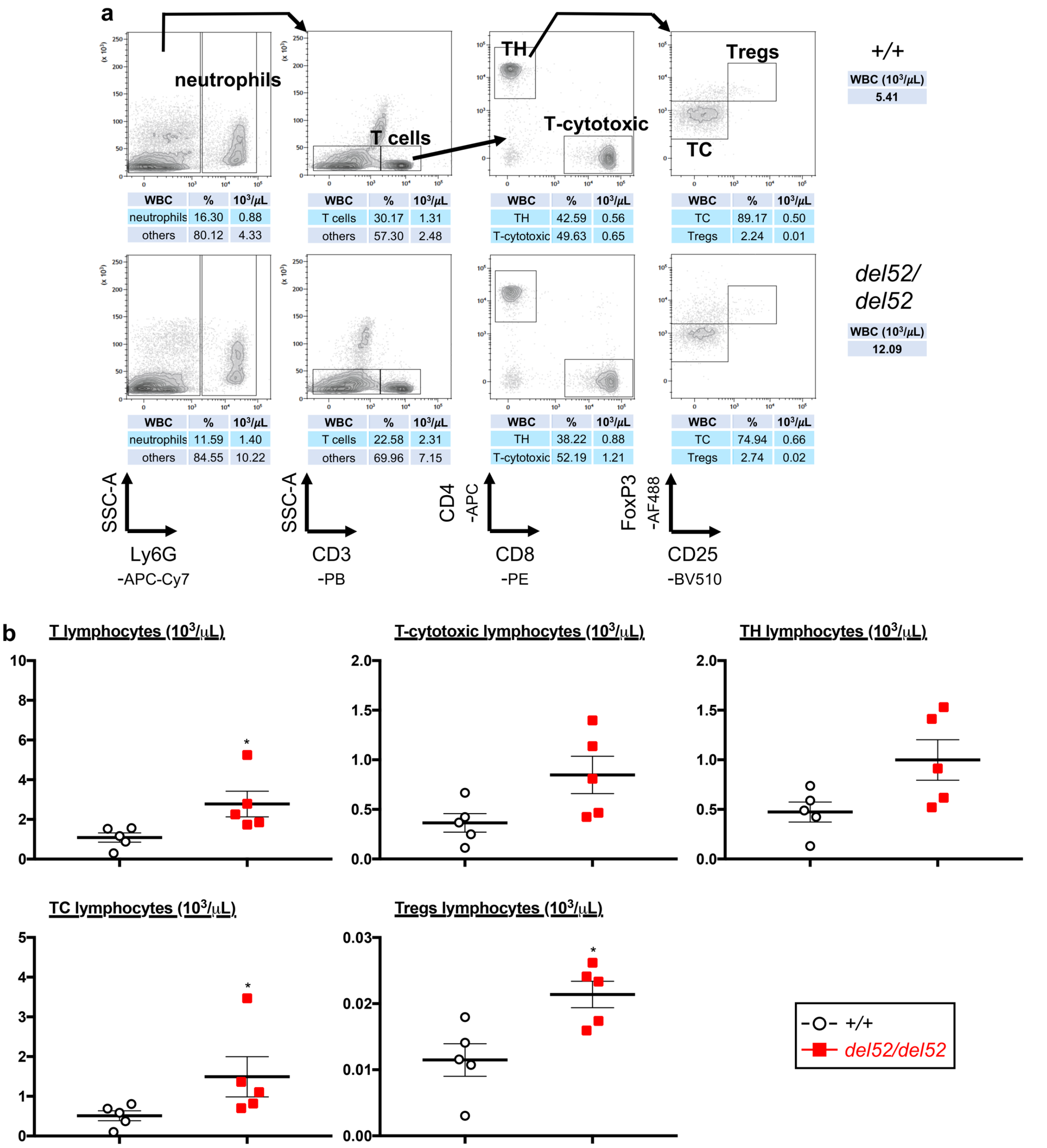

WBC	%	$10^3/\mu L$
neutrophils	16.30	0.88
others	80.12	4.33

WBC	%	$10^3/\mu L$
T cells	30.17	1.31
others	57.30	2.48

WBC	%	$10^3/\mu L$
TH	42.59	0.56
T-cytotoxic	49.63	0.65

WBC	%	$10^3/\mu L$
TC	89.17	0.50
Tregs	2.24	0.01

WBC	%	$10^3/\mu L$
neutrophils	11.59	1.40
others	84.55	10.22

WBC	%	$10^3/\mu L$
T cells	22.58	2.31
others	69.96	7.15

WBC	%	$10^3/\mu L$
TH	38.22	0.88
T-cytotoxic	52.19	1.21

WBC	%	$10^3/\mu L$
TC	74.94	0.66
Tregs	2.74	0.02

Supplementary Fig. 3. Characterization of T lymphocyte sub-populations of *CALR del52* KI mice. a Gating strategy used to analyze T lymphocyte composition in WBC by flow cytometry from blood of ≥10-month-old +/+ (open circles) and *del52/del52* (red squares) KI mice. Absolute numbers of T-cytotoxic ($CD3^+CD8^+$), T-helper (TH, $CD3^+CD4^+$), regulatory $CD4^+$ T (Tregs, $CD3^+CD4^+CD8^-FoxP3^+CD25^{+/-}$ and conventional $CD4^+$ T cells (TC, $CD3^+CD4^+CD8^-FoxP3^-CD25^-$) were determined by flow cytometry and **b** expressed as

means±SEM (n=5 independent mice). Significance was calculated with two-sided parametric t-test (*p=0.0393 for T, 0.014 for Tregs lymphocytes) and a two-sided nonparametric Mann-Whitney test (only for TC cells) with *p=0.0159. Source data are provided as a Source Data file.

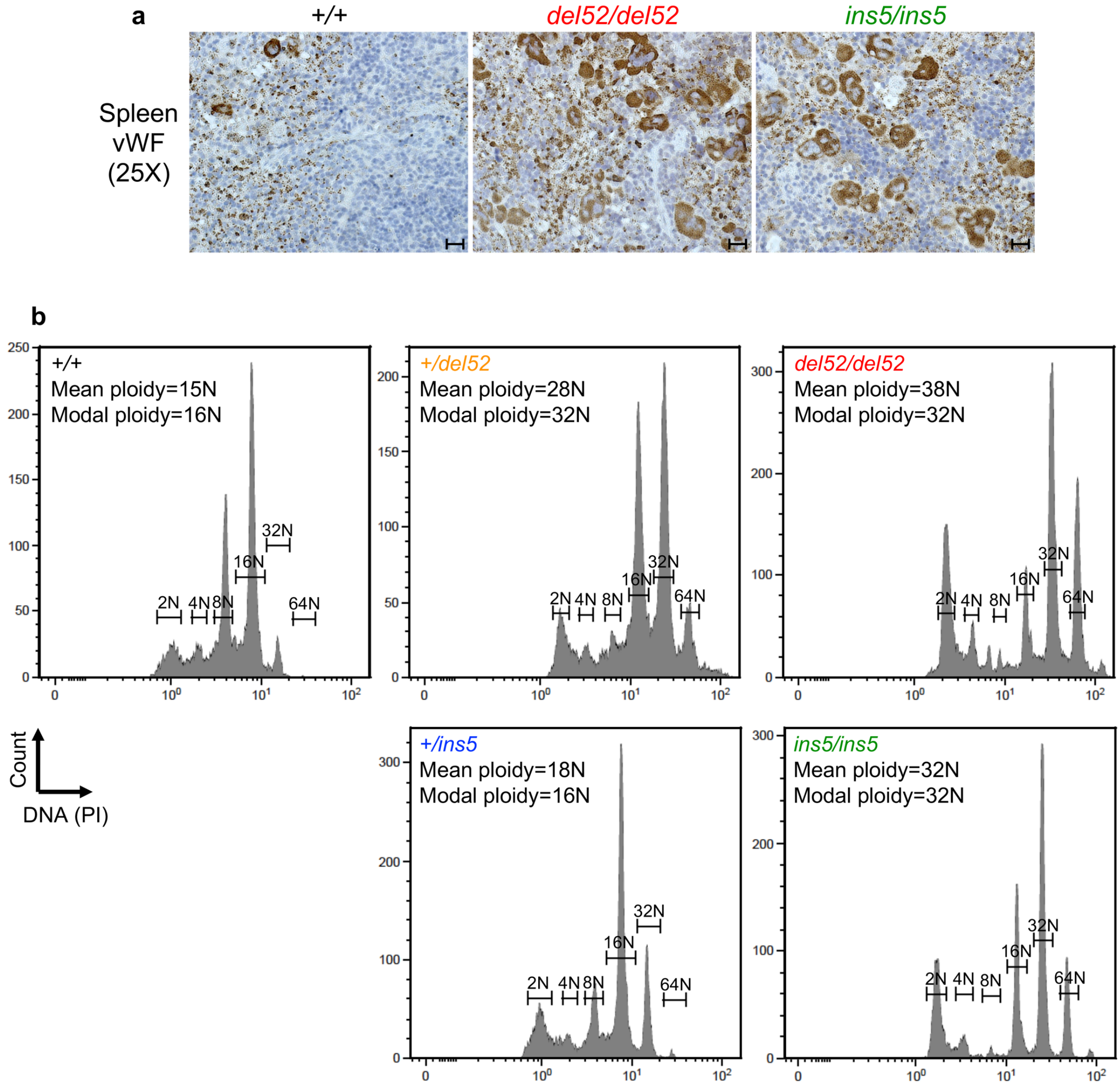

Supplementary Fig. 4. Analysis of BM and spleen MKs from *CALR del52* and *CALR ins5* KI mice. a Spleen MKs were immunostained for vWF. Images were obtained using a DM2000 Leica microscope and a DFC300FX Leica camera with Leica Application Suite v.2.5,OR1 acquisition software (40X magnification). Scale bars represent 50 µm. Similar results were obtained in analyzing at least 3 independent mice for each genotype. **b** Data illustrate flow cytometry analysis of mean (≥8N) and modal ploidies of MKs from ≥10 months post-tamoxifen KI mouse BM stained with anti-CD41 and anti-CD42 antibodies and propidium iodide (PI) labeling of DNA.

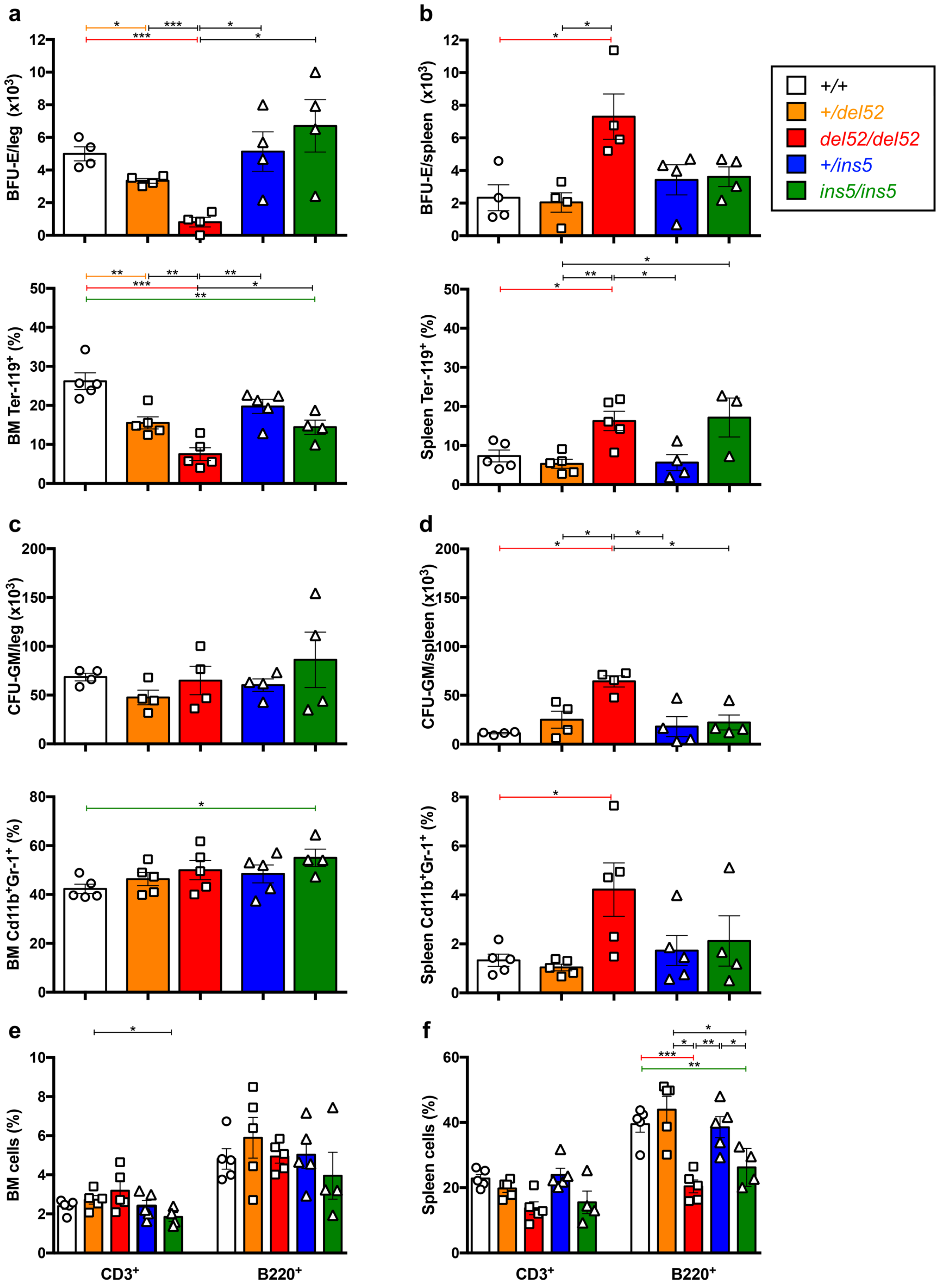

Supplementary Fig. 5. Effect of *CALR del52* and *CALR ins5* on myeloid progenitors. Percentages of erythroid (Ter-119^{+}) and granulocyte (Cd11b^{+}Gr-1^{+}) precursors with numbers of erythroid (BFU-E) and granulomonocytic (CFU-GM) progenitors were analyzed in **a**, **c** BM per leg and **b**, **d** entire spleen of ≥10 months post-tamoxifen *+/del52*, *del52/del52*, *+/ins5* and

ins5/ins5 compared to +/+ mice. Percentages of T (CD3$^+$) and B (B220$^+$) lymphocytes were analyzed in **e** BM and **f** spleen. Data are presented as means±SEM with n=5 individual mice except n=4 for *in5/ins5* genotype when analyzing Ter-119$^+$, Cd11b$^+$Gr-1+ and CD3$^+$ or B220$^+$ and n=4 for all genotypes in BFU-E and CFU-GM analyses. In the figure, white is used for +/+, orange for *+/del52*, red for *del52/del52*, blue for *+/ins5* and green for *ins5/ins5* genotypes. Significance was calculated with a two-sided parametric t-test, and a two-sided nonparametric Mann-Whitney test (only for CFU-GM/spleen). In a, *p=0.0112 except 0.0133 for *del52/del52 vs. +/ins5*; ***p=0.0002 *(+/+ vs. del52/del52*), 0.0003 (*+/del52 vs. del52/del52*) in BM BFU-E analysis and *=0.0252; **p=0.0038 (*+/+ vs. +/del52*), 0.0049 (*+/+ vs. ins5/ins5*), 0.0072 (*+/del52 vs. del52/del52*), 0.001 (*del52/del52 vs. +/ins5*), ***p=0.0001 in BM Ter-119$^+$ analysis. In b, *p=0.021 (*+/+ vs. del52/del52*), 0.0131 (*+/del52 vs. del52/del52*) in spleen BFU-E analysis and *p=0.0148 (*+/+ vs. del52/del52*), 0.0237 (*+/del52 vs. ins5/ins5*), 0.0153 (*del52/del52 vs. +/ins5*); **p=0.038 in spleen Ter-119$^+$ analysis. In c, *p=0.0125. In d, *p=0.0286 in BM GFU-GM analysis and *p=0.0323 in spleen Ter-119$^+$ analysis. In e, *p=0.0281. In f, *p=0.0295 (*+/del52 vs. del52/del52*), 0.0125 (*+/del52 vs. ins5/ins5*), 0.0283 (*+/ins5 vs. ins5/ins5*); **p=0.0099 (*+/+ vs. ins5/ins5*), 0.0014 (*del52/del52 vs. +/ins5*); ***p=0.0003. Source data are provided as a Source Data file.

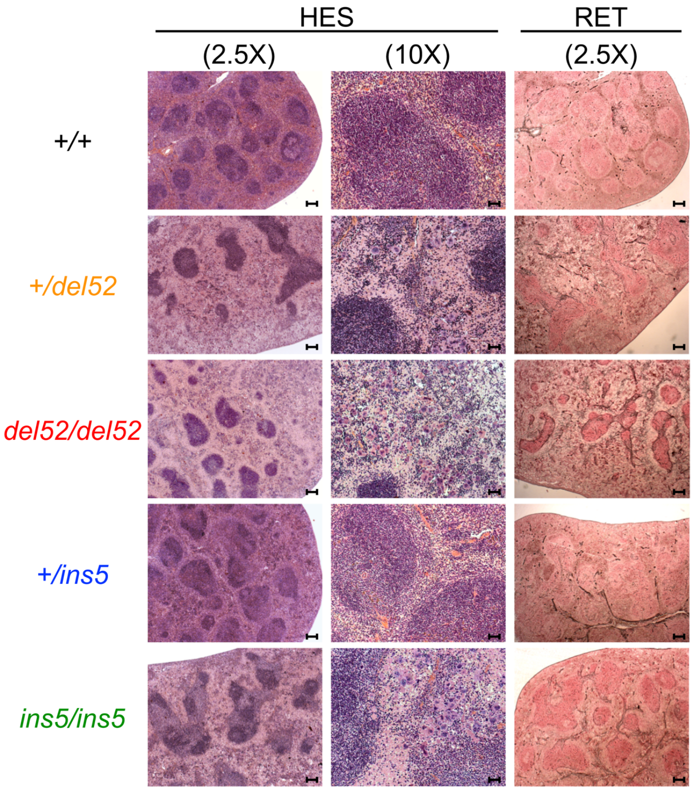

Supplementary Fig. 6. Spleen structure and fibrosis of KI mice. Images show low magnifications (2.5X and 10X) of the histopathology of spleen of ≥10 months post-tamoxifen mice stained with HES and RET. Images were obtained using a DM2000 Leica microscope and a DFC300FX Leica camera with Leica Application Suite v.2.5,OR1 acquisition software. Scale bars represent 50 µm. Similar results were obtained in analyzing at least 3 independent mice for each genotype.

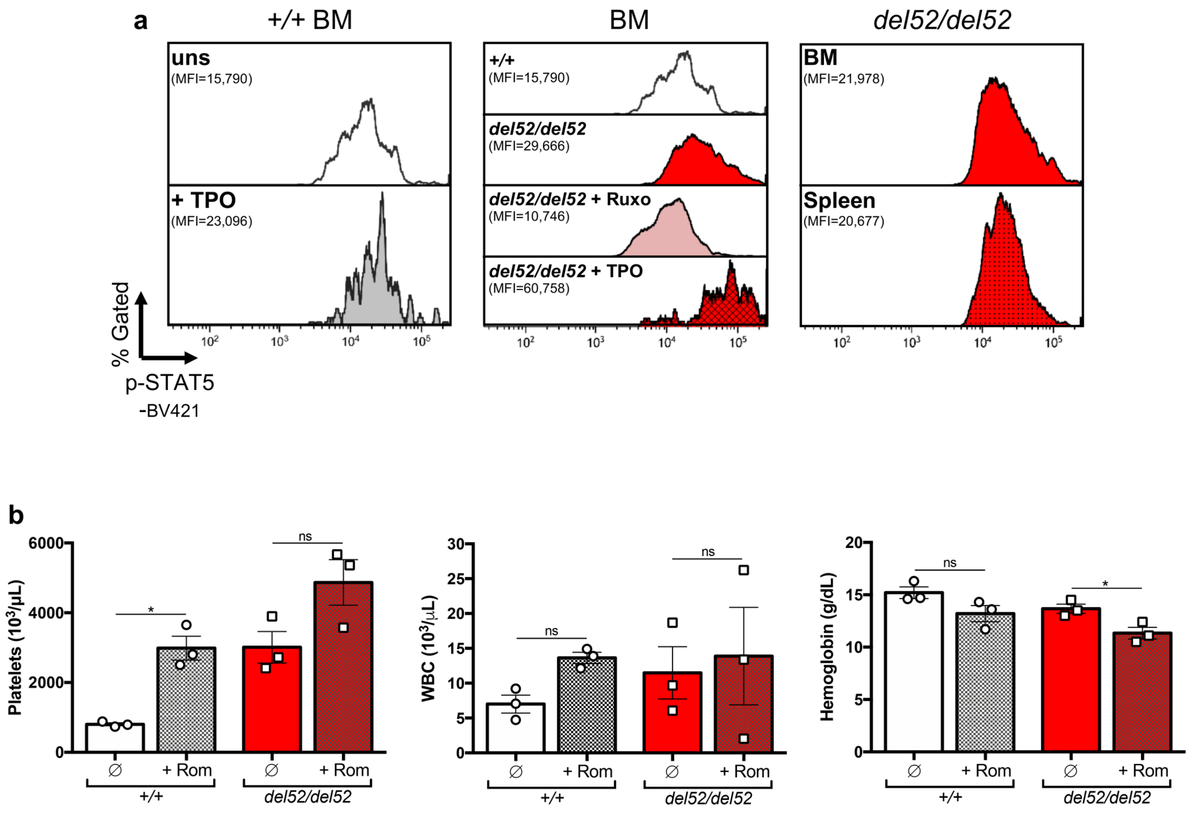

Supplementary Fig. 7. Effect of MPL signaling level and JAK2/STAT5 activation in BM and spleen MKs in the intensity of phenotype developed by KI mice. a Phospho-STAT5 (p-STAT5) was assessed by phosphoflow assay in BM and spleen MKs of ≥10 months post-tamoxifen *del52/del52* KI mice and wt (+/+) littermate, without (unstimulated, uns) or with TPO stimulation (+ TPO) or treatment with the JAK1/2 inhibitor ruxolitinib (+ Ruxo), as indicated. Graphs show the mean fluorescence intensity (MFI) for a representative experiment. **b** Comparison of platelet, WBC and hemoglobin levels in 1 month post-tamoxifen *del52/del52* (red) KI mice and wt (+/+) littermates (white) before (∅, solid histograms) and after the last injection of romiplostim (+ Rom, hatched histograms). Data are expressed as means±SEM (n=3 independent mice) and significance of + Rom to ∅ condition was assessed using a parametric paired two-sided t-test (*p=0.0263 (Platelets) and 0.0204 (Hemoglobin); ns: not significant). Source data are provided as a Source Data file.

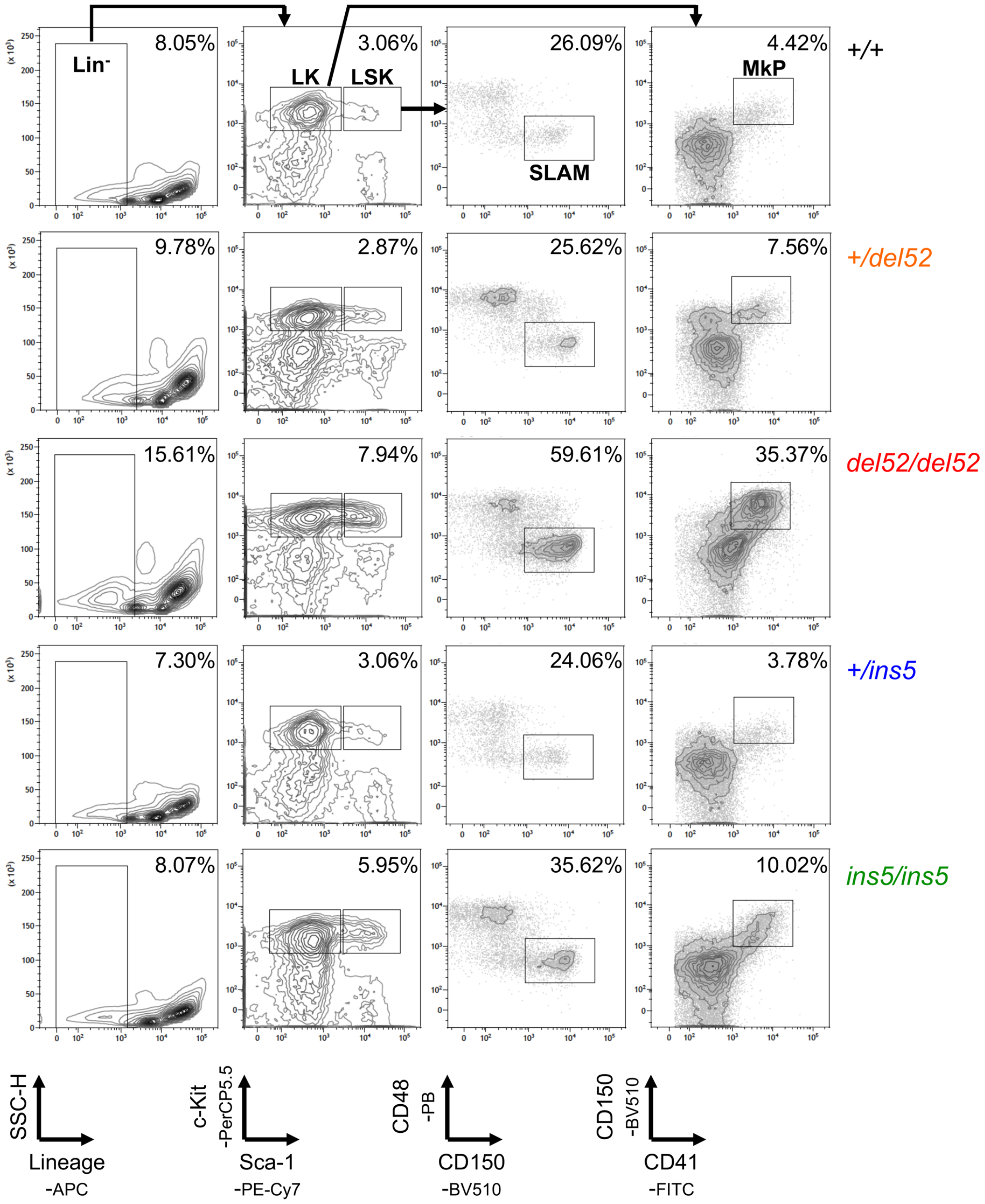

Supplementary Fig. 8. Cell-surface phenotyping of *CALR del52* and *CALR ins5* KI mouse BM cells. BM of mice ≥10 months post-tamoxifen induction was labeled with a combination of Abs to analyzed the Lin$^-$, hematopoietic stem cell-enriched Lin$^-$Sca-1$^+$c-Kit$^+$ (LSK) and LSKCD48$^-$CD150$^+$ (SLAM) cell populations and the MK progenitor LKSca-1$^-$CD150$^+$CD41$^+$ (MkP) in wild-type (+/+), heterozygous *(+/del52* and *+/ins5*) and homozygous (*del52/del52* and *ins5/ins5*). Data shown are representative of a typical experiment.

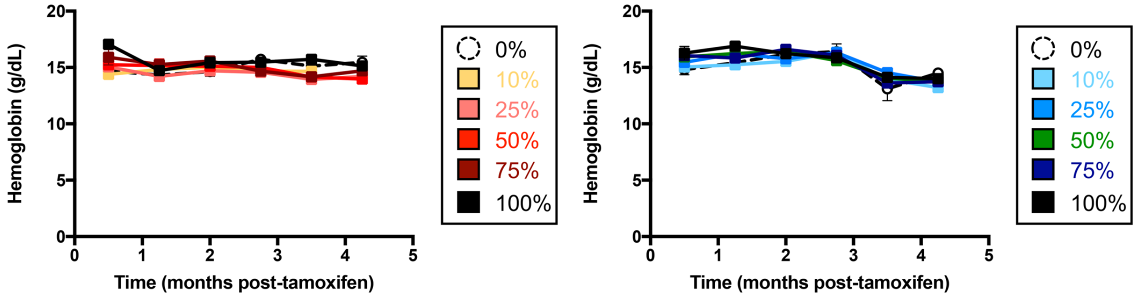

Supplementary Fig. 9. Hemoglobin analysis of competitive transplantation models. Lethally-irradiates recipient mice were engrafted with various ratios of *del52/del52* (left panel, yellow, pink, red, brown and black squares and connecting solid lines) or *ins5/ins5* (right panel, turquoise, blue, green, dark blue and black squares and connecting solid lines) BM cells. After induction by tamoxifen, hemoglobin was measured. Data are the means±SEM (n=3-5 individual mice, with n varying depending on time of sampling during the course of the experiment and on the group defined as the percentage of engrafted homozygous cells, as indicated in the Source Data file). There was no significant difference compared to the 0% condition as determined using ANOVA with Dunnett's multiple comparison test for *ins5/ins5* and a nonparametric Kruskal-Wallis's multiple comparison test for *del52/del52* data. Source data are provided as a Source Data file.

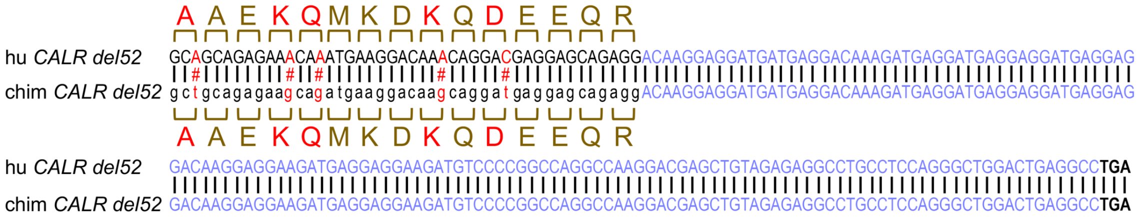

Supplementary Fig. 10. Alignment of hu *CALR del52* and chim *CALR del52* exon 9 sequences. Human sequence is depicted in upper cases and murine sequence in lower cases with the purple upper cases sequence encoding the human mutated C-terminal tail. Red fonts in the DNA sequences indicate the mismatches between the human and murine DNAs and are all located in the silent position of the codons, giving rise to similar amino acids in red. Thus, the exact same chimeric CALR protein is generated with either replacing the entire murine exon 9 with the human mutated exon 9 (Li *et al.*) or adding the human sequence encoding the modified C-terminal tail after the murine *del52* mutation (our strategy).

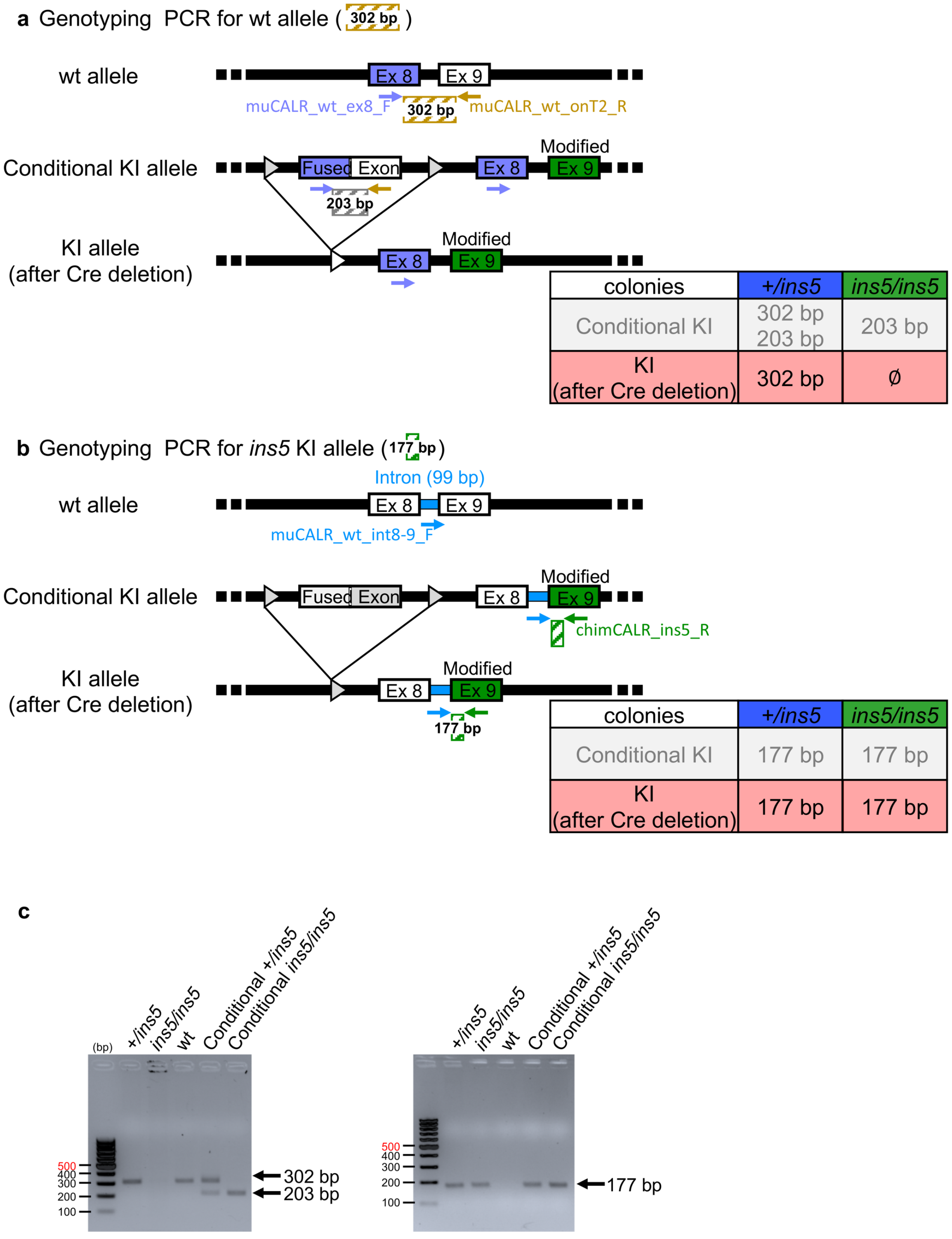

Supplementary Fig. 11. Strategy for genotyping *+/ins5* and *ins5/ins5* colonies from competitive *ins5* KI transplantation model. a The pair of primers muCALR_wt_ex8_F and muCALR_wt_onT2_R was used to detect the wt mouse *Calr* allele (302 bp). The pair of primers can also amplify a 203 bp product on the conditional KI allele (before Cre

recombination induced by tamoxifen). **b** The pair of primers muCALR_wt_int8-9_F and chimCALR_ins5_R was used to identify the *ins5* KI allele by amplifying a product on the modified exon 9 encoding the human mutated C-terminal tail (177 bp) before or after Cre recombination. The 2 tables in **a** and **b** give a summary of the product sizes obtained after PCR using DNA from conditional (grey background) and tamoxifen-induced (after Cre deletion, light red background) heterozygous *(+/ins5*) and homozygous (*ins5/ins5*) KI colonies. **c** Examples of PCR products obtained using the 2 sets of primers for the wt (left panel) and the KI (right panel) alleles. The absence of the 203 bp band helps to validate the efficacy of tamoxifen-induced Cre deletion. The similar pair of gels was resolved to genotype an average of 58 colonies/mouse with n=3 independent mice.
